# A 5-year review of research ethics applications in a tertiary health and educational institution in Nigeria

**DOI:** 10.4314/ahs.v23i3.85

**Published:** 2023-09

**Authors:** Dennis Amajuoyi Ndububa, Akinjide Olurotimi Ogundokun, Oluwagbemiga Oluwole Ayoola, Adebanjo Babalola Adeyemi, Rahman Ayodele Bolarinwa, Taiwo Olumuyiwa Ogundipe, Abdulkadir Ayo Salako, Aaron Oladiipo Aboderin, Olusegun Temitope Afolabi, Anthony Taiwo Adenekan, Ige Oluwatosin Taiwo, Oluwabanke Gold Akanbi

**Affiliations:** 1 Department of Medicine, Faculty of Clinical Sciences, College of Health Sciences, Obafemi Awolowo University (OAU), Ile-Ife, Nigeria; 2 Department of Family Medicine, Obafemi Awolowo University Teaching Hospitals Complex (OAUTHC), Ile-Ife, Nigeria; 3 Department of Radiology, Faculty of Clinical Sciences, College of Health Sciences, OAU, Ile-Ife, Nigeria; 4 Department of Obstetrics, Gynaecology & Perinatology, Faculty of Clinical Sciences, College of Health Sciences, OAU, Ile-Ife, Nigeria; 5 Department of Haematology, Faculty of Clinical Sciences, College of Health Sciences, OAU, Ile-Ife, Nigeria; 6 Department of Pharmacy, OAUTHC, Ile-Ife, Nigeria; 7 Department of Surgery, Faculty of Clinical Sciences, College of Health Sciences, OAU, Ile-Ife, Nigeria; 8 Department of Medical Microbiology & Parasitology, Faculty of Basic Medical Sciences, OAU, Ile-Ife, Nigeria; 9 Department of Community Health, Faculty of Clinical Sciences, College of Health Sciences, OAU, Ile-Ife, Nigeria; 10 Department of Anaesthesia & Intensive Care, Faculty of Clinical Sciences, College of Health Sciences, OAU, Ile-Ife, Nigeria; 11 Hospital Legal Unit, OAUTHC, Ile-Ife, Nigeria; 12 Department of Clinical Services & Training, OAUTHC, Ile-Ife, Nigeria

**Keywords:** Research ethics applications, study sponsorship

## Abstract

**Background:**

African contribution to global research output is said to be low. Poor funding and poor skills in grant writing have been suggested as important factors for this situation.

**Objectives:**

Applications for research ethics clearance in a hospital were reviewed to have an overview of the planned studies and the proportion of them that attracted national and international funding.

**Methods:**

A review of all applications for ethical clearance received by the institutional review board of a university teaching hospital at Ile-Ife, Nigeria, from 2016 to 2020. They were analysed according to study nature, scope, purpose, and sponsorship using descriptive statistics presented as frequency tables and charts.

**Results:**

A total of 878 applications were reviewed. There were 803 (91.5%) applications for local, 45 (5.1%) for national multicentre, and 30 (3.4%) for international multicentre studies. Applications for medical fellowship were 352 (40.0%) while 208 (23.8%) were from academic staff for non-degree research. There were 610 (69.5%) applications for self-sponsored studies. Only 18 (2.0%) and 26 (3.0%) received sponsorship from national and international donor agencies, respectively.

**Conclusions:**

Local studies formed the bulk of the submissions for ethics clearance. National and international donor funding of research is abysmally low in this Nigerian tertiary institution studied.

## Introduction

Research is a diligent study of a subject or phenomenon with a view to discovering new facts or providing new insights. Many countries, especially of the developed world, have made giant strides socio-economically and technologically through research breakthroughs. Very low priority is given to scientific research by countries of the developing world as there are seemingly more pressing issues such as food, shelter, infrastructure, and security^1^. It is no wonder, therefore, that only 1% of global research output is said to be contributed from Africa^2^.

One important function of tertiary institutions such as universities and teaching/specialist hospitals is the conduct of research^3^. However, it has been observed that a major motivation for research in institutions in Africa is the aspiration to attain academic qualifications^4^. The conduct of productive and impactful research requires adequate funding and political will from governments and policy makers. The absence of a clearcut philosophy of national development, political instability and government bureaucracy and corruption involved in accessing approved funds where available are some of the reasons given for the low research output from Africa^5^. We therefore reviewed applications for research ethics clearance from academicians and postgraduate doctors and students and undergraduates in a Nigerian tertiary health and educational institution with a view to determining the extent to which these factors may have affected the nature, scope, and funding of their planned studies.

## Methods

All applications for research ethics clearance submitted to the Ethics and Research Committee of the Obafemi Awolowo University Teaching Hospitals Complex, Ile-Ife, Nigeria, between January 2016 and December 2020 were reviewed retrospectively. This local Institutional Review Board (IRB) has been serving the researchers in the hospital, its sister institution (Obafemi Awolowo University), and other health and academic institutions in the country using the hospital as a study site since its inception about 20 years ago. Its trained 17-member team led by a chairperson, and representing various disciplines in Medicine and community interests, meets once a month to do a conference review of all research ethics applications submitted to it after individual expert review. This local IRB is under the supervisory control of the National Health Research Ethics Committee (NHREC) of the Federal Ministry of Health, Abuja, Nigeria.

Applications from undergraduates, postgraduate students and doctors and academic/clinical staff of these institutions are first assigned to members of the committee based on their area of specialization. This is followed by a conference review of the applications at the monthly statutory meeting of the committee. Occasionally, an application is sent for review to a non-member with expertise in an area lacking among the committee members. For this review, four major aspects of the applications were studied, namely, (1) the nature of the proposed study, (2) the study scope or coverage, (3) the study purpose, and (4) the sponsorship. Under the study nature, the specialties, and disciplines from which the applications emanated were examined while the spread of the research activity was examined under the study scope. Under the study purpose, the research intent was examined, whether for degree or non-degree purposes. The applications were also scrutinized to determine what proportion of them was grant-aided by institutional, national, or international bodies and what proportion was by self-sponsorship. The results were analysed using descriptive statistics presented as frequency tables and charts.

## Results

A total of 886 applications for research ethics clearance were received during the period under review. However, 8 of the applications contained incomplete information and so were dropped leaving 878 applications for analysis. The specialty with the highest number of applications was general surgery (155, 17.7%) followed by medical rehabilitation (103, 11.7%), nursing (102, 11.6%), internal medicine (96, 10.9%), and laboratory medicine (91, 10.4%). However, most of the applications from medical rehabilitation (87, 84.5%) and nursing (59, 58%) were undergraduate applications for first degree purposes (Table 1). Medical microbiology & parasitology topped the list of applications from Laboratory Medicine (42, 46%).

**Table 1:** Research Ethics Applications According to Study Specialty (n=878)

Study Specialty	No. (%)
General Surgery	155 (17.7)
θMedical Rehabilitation	103 (11.7)
ΩNursing	102 (11.6)
Internal Medicine	96 (10.9)
*Laboratory Medicine	91 (10.4)
Obstetrics & Gynaecology	43 (4.9)
Radiology	37 (4.2)
Paediatrics	34 (3.9)
Dentistry	32 (3.6)
Pharmacy	27 (3.1)
#Surgical Specialties	27 (3.1)
Family Medicine	23 (2.6)
Dermatology	16 (1.8)
Mental Health	16 (1.8)
ϕOthers	76 (8.7)

θ Out of these 103, 87 (84.5%) were undergraduate applications

Ω Out of these 102, 59 (58%) were undergraduate applications

* Chemical Pathology (7), Haematology (26), Medical Microbiology & Parasitology (42), Morbid Anatomy & Forensic Medicine (15) + 1 entry rendered simply as Laboratory Medicine

# Orthopaedic Surgery & Traumatology (13), Ophthalmology (11), Otorhinolaryngology (2), Paediatric Surgery (1)

ϕ Anaesthesia (13), Sociology & Anthropology (8), Community Health (7), Computer Science (6), Engineering (6) Psychology (3), Physiology (3), English/Literary Studies (3), Zoology (3), Nutrition/Dietetics (2), Physical & Health Education (2), Political Science (2), Urban & Regional Planning/Estate Management (2), Ecology/Geography (2), Health Information Management (2), Anatomy, Architecture, Biological Sciences, Chemistry, Communication & General Study, Economics, History, Library/Information Science, Mathematics, Public Administration, Religious Studies, Science Policy & Innovation (1 each)

Overwhelming majority (803, 91.5%) of the applications were for local studies to be conducted within the immediate vicinity of the institution of the principal investigators. Forty-five (5.1%) applications were in respect of multicentre studies involving institutions and locations in other parts of the country while 30 (3.4%) applications were for international multicentre studies (Table 2).

**Table 2:** Research Ethics Applications According to Study Scope (n=878)

Scope of Study	No. (%)
Local	803 (91.5)
National Multicentre	45 (5.1)
International Multicentre	30 (3.4)

The largest number of applications was for the purpose of obtaining Medical Fellowship (352, 40.1%) by postgraduate medical doctors undergoing their Residency Training Programme. This was followed by applications from academic and/or clinical staff (208, 23.7%) undertaking research as part of their responsibilities. Applications for Bachelor, Master and PhD degrees were 171 (19.5%), 110 (12.5%), and 37 (4.2%), respectively (Table 3).

**Table 3:** Research Ethics Applications According to Purpose of Study (n=878)

Purpose of Study	No. (%)
A. Degree Purposes	
1. Undergraduate (B. Sc.)	171 (19.5)
2. Postgraduate (M.Sc./MPH)	110 (12.5)
3. Postgraduate (PhD)	37 (4.2)
B. Medical Fellowship	352 (40.1)
C. Not for Degree or Fellowship	208 (23.7)

Out of the 878 applications analysed, 610 (69.5%) were for studies to be sponsored by the applicants themselves. The hospital was to provide part-sponsorship for 216 (24.6%) applications while 8 (0.9%) applications were to receive full sponsorship from the hospital. Seven (0.8%) and 11 (1.2%) applications provided evidence of sponsorship of their proposed studies from the Tertiary Education Trust Fund (TET Fund) and non-governmental organisations (NGO) within the country, respectively. Twenty-six (3.0%) applications had the sponsorship backing of international grants/donor agencies (Table 4).

**Table 4:** Research Ethics Applications According to Study Sponsorship (n=878)

Study Sponsorship	No. (%)
Self Only	610 (69.5)
Self + Hospital	216 (24.6)
Hospital Only	8 (0.9)
University *TET Fund	7 (0.8)
National #NGO	11 (1.2)
International Grants/Donor Agency	26 (3.0)

* Tertiary Education Trust

# Non-Governmental Organisation

## Discussion

This study shows that an average of almost 180 applications per annum were submitted for research ethics clearance by the IRB in the period under review. Even though the bulk of the applications were, understandably, from the medical sciences, an appreciable number of them were also submitted from the social sciences, engineering, information, and communication technology (ICT), environment and development studies. This development, if replicated in other institutions in the country, is bound to positively impact on the ethical standards of research emanating from Nigeria. This is because a multi-disciplinary approach to research ethics administration, especially where a study in a particular discipline has the potential to impact another, will foster understanding among researchers from different backgrounds and broaden the scope of ethical issues in scientific research. This underscores the call for the strengthening of research ethics committees (RECs) and the observation that the RECs in Africa are well organized and functional^6^,^7^.

There is no doubt that research is vital for the growth and development of any country. It is a veritable instrument for the transformation of societies. Advances and breakthroughs have been recorded in Medicine, and in other disciplines, through research. However, research requires motivation and funding for the researcher and special attention and political will from the policy maker. Research output from the developing countries of the world, especially Africa, is regarded to be among the lowest^2^. Several challenges have been identified to be responsible for this situation. These challenges that make scientific research take a back seat in Africa include more pressing priorities such as hunger, epidemics, shelter, and insecurity with its attendant refugee crisis1. Others are little or no state funding and decayed infrastructure^3^,^8^.

The specialties or disciplines appear well represented in this study as shown by applications submitted for research ethics approval. However, over 90% of them were for local studies, which were mainly hospital-based. More far-reaching conclusions and authoritative findings usually necessitate the conduct of community-based, national, or international multicentre studies. Regrettably, in this study less than 10% of the applications had their proposed studies in this category. This is obviously due to limited resources available to the researcher which then makes his main motivation for research to be the quest for promotion or the acquisition of a degree^4^. This was attested to by some Nigerian medical specialists who admitted to being involved in low budget research projects that hardly produced significant impact and outcomes^9^.

Majority (40.1%) of the applications were for the purpose of obtaining the Medical Fellowship necessary for appointment as Specialists/Consultants and as lecturers in the medical school. This category of applicants are postgraduate doctors or Residents cutting their teeth in scientific research. Applications from academic and clinical staff writing to contribute to knowledge and medical practice and for promotion came a distant second (23.7%). This latter category of researchers are mainly medical Consultants in the hospital who also hold lectureship positions in the university. Laboratory support for them is grossly insufficient as research laboratories are often lacking and where they exist, functional equipment and skilled technicians are absent1. These researchers in Clinical Medicine are also adversely affected by lack of availability of constant high-speed internet, heavy clinical workload, lack of incentives, and brain drain^1^,^4^,^5^,^10^,^11^.

These factors, no doubt, impact negatively on their research productivity. The applications from candidates pursuing Master and Doctorate degrees altogether constituted 16.7%. This is understandably low as these were only postgraduate students from the sister university whose research required hospital patients or staff as study participants.

This review showed that a great majority (69.5%) of the applicants for ethical clearance were on self-sponsorship for their research. This, no doubt, would place great constraints on the scope, quality, and impact of research. Research that will produce authoritative findings require funds for such resources as personnel (emoluments for researcher and hired assistants), laboratory equipment and reagents, logistics and incentives among others. Lack of corporate or state funding has been severally highlighted as a bane of research in the developing countries^3^,^4^,^8^,^10^,^12^.

Only about a quarter of the postgraduate doctors in the employ of the hospital whose ethics applications were reviewed were supported as part of the hospital policy of part-sponsorship of Resident Doctors preparing for their Medical Fellowship. The proportion of applicants that were able to secure grants from national and international donor agencies was abysmally low (5%). The Tertiary Education Trust Fund (TET Fund) was established by the government of Nigeria to fund tertiary education in the country with tax money contributed by companies. Part of the TET Fund is earmarked for research, but concern has been expressed as to whether research is prioritized by the Nigerian government and whether TET Fund is truly functional regarding research funding^11^.

However, to have a donor agency is one thing, it is another thing to be able to access fund from the agency^5^. The need for education in grant writing has been emphasized as an aid to accessing funds and for research capacity strengthening generally^13^. Training of early-career researchers (both physical and online) in Africa in research development skills and grant productivity is, therefore, highly desirale^14^,^15^. The outcome of this training will, no doubt, result in the generation of a great number of proposals which has been touted as the surest way to win grants from donor agencies^16^. However, an assessment of the knowledge of the applicants about grant writing was not done in this study. It was not possible, therefore, to determine how it may have contributed to the level of funding support found in this study. Steps to improve both local and international funding of research in Africa will obviously include the prioritization of research by governments, increased collaboration among researchers across borders in the continent, the institution of research capacity building courses and involvement of the private sector in research funding and demand for research results^1^,^2^,^4^,^11^.

In conclusion, this review of research ethics applications from a tertiary hospital in Southwest Nigeria has shown that an overwhelming majority of the applications were for local studies limited to the immediate vicinity of the hospital and mostly sponsored by the researchers themselves with abysmally low funding support from both national and international funding agencies.
